# Labels, Rationality, and the Chemistry of the Mind: Moors in Historical Context

**DOI:** 10.1080/1047840X.2017.1256605

**Published:** 2017-02-26

**Authors:** Thomas Dixon

**Affiliations:** ^a^ Centre for the History of the Emotions, Queen Mary University of London, London, United Kingdom

I am grateful to the editor of *Psychological Inquiry* for the opportunity to read and reflect on Professor Agnes Moors's (this issue) fascinating target article, which offers an extremely helpful analysis of existing theories of emotion, as well as some bold proposals for a new approach. I am not qualified to comment on the empirical and theoretical merits of Moors's arguments from the point of view of contemporary psychology. Instead I offer here some thoughts inspired by reading Moors's article, from my perspective as a historian of emotions and philosophy.

The history of emotions, as a field, tends toward an appreciation of the cultural specificity and diversity rather than the universality of human emotional life (Frevert, 2011; Plamper, 2015; Watt Smith, 2015). As such, it is common to find that historians of emotions are more sympathetic to constructionist accounts of human emotions and sceptical of the claims of “basic emotion” and “affect program” theories (Leys, 2010). In what follows I comment first on Moors's thoughts about psychological categories, then on the rationality or otherwise of emotional behaviors, before ending with some thoughts about whether the composition of an emotional episode is more like a physical aggregate or a chemical compound.

In my previous work I have suggested that the Scottish philosopher, physician, and poet Professor Thomas Brown (1778–1820) was the inventor of “the emotions” as a systematic psychological category. In his widely read *Lectures on the Philosophy of the Human Mind*, originally delivered to students in Edinburgh and subsequently published in 1820, Brown (2010) noted, “The exact meaning of the term *emotion*, it is difficult to state in any form of words” (pp. 145). He went on, “Perhaps, if any definition of them be possible, they may be defined to be vivid feelings, arising immediately from the consideration of objects, perceived, or remembered, or imagined, or from other prior emotions” (Brown, 2010, pp. 145–146).

As we approach the bicentenary of the publication of Brown's lectures, perhaps it is finally time for psychology to give up on Brown's aspiration to provide a coherent theoretical account of those vivid but ill-defined feelings known as “the emotions.” Agnes Moors's article certainly seems to imply as much.

Moors suggests that not only “emotion” and “the emotions” but also all “vernacular emotion subsets,” that is, particular emotion words like “anger,” “fear,” or “surprise,” fail the tests of similarity and fruitfulness, which mark out genuinely useful scientific categories. For Moors, in other words, a satisfactory theory of the emotions cannot be found, because “the emotions” do not exist as a coherent psychological domain, or “scientific set.” As far as I understand her argument, Moors wants to suggest, in agreement with psychological construction theory, that an emotional episode becomes an emotional episode only when the person experiencing it labels it as such. It is labeling, and not any underlying biological or psychological structure, that qualifies an episode for inclusion in the category of “emotion.”

Moors, then, has theoretical psychological reasons, and views about scientific explanation, that lead her to argue that “emotion” or “the emotions” are unsatisfactory psychological categories. There are other reasons to make similar arguments. I have done so myself, for instance, on the basis of intellectual history (Dixon, 2003, 2012). There is a difference between “emotion” and “the emotions” as historical psychological terms. “Emotion” in the singular can function like “feeling” or “affect” as a general abstraction for a mode of agitated felt experience, and in fact it has been used this way since the 18th century. “The emotions,” on the other hand, appeared as a discrete psychological category only in the mid-19th century, aiming to bring together states including love, hatred, joy, sadness, fear, anger, and so on, under a unifying theoretical umbrella. Moors could draw upon research in both linguistics and history for further support for the idea that the categorization of experiences using the language of “emotion” is a feature of post-1850 English-language psychology (both vernacular and scientific), which does not map directly onto the categories of other times, languages, and cultures (Frevert et al., 2014; Wassmann, 2016; Wierzbicka, 1999, 2010).

I lack the disciplinary expertise to appreciate the significance of some other linguistic distinctions that Moors sets out to make but, on the face of it, I could not see why she considered “perception,” “appraisal,” and “categorization” unhelpful, and interchangeable, terms while her favored alternative language of “input” and “output,” “content,” “representation,” and “extraction mechanisms” were supposed to be more objective, neutral, and scientific. All of these terms, both those Moors rejects and those she adopts, seem comparably neutral and formal or, to look at it from the opposite direction, equally open to skeptical deconstruction as unstable fictions by those inclined to doubt their value. I can imagine a critic of Moors's approach to “emotion” similarly asking whether her favored categories of “behavior” and “experience” are ultimately more coherent and robust than “emotion” as scientific categories. One of the strengths of the kind of scientific and philosophical skepticism deployed by Moors is that it can be turned on almost any theoretical abstraction to good effect. I suspect that the quest for a set of psychological terms that is neutral, scientific, and immune to such criticisms, whatever its value, will probably never end.

On the theoretical model favored by Moors, emotional episodes are to be examined alongside nonemotional episodes in terms of the states of consciousness and types of behavior they involve. In the domain of behavior, Moors suggests that a goal-directed theoretical approach can show that although emotional behaviors may have an irrational flavor to them, they are not in fact irrational in nature. Moors refers to the commonly held belief (common, at least, outside the realm of academic experts on emotions) that emotional behavior is too fast and automatic, and sometimes too counterproductive, to be considered rational. She also suggests a couple of examples of apparently harmful emotional behavior—fighting in the context of a relationship and avoiding eye contact in a job interview. Moors's proposal is that, if considered goal-directed behaviors, such episodes can seem less irrational. Agents have multiple goals, and a form of behavior that seems harmful in relation to achieving one goal (maintaining a relationship) might be conducive to the attainment of another goal (maintaining social status) that is of more importance to the subject, more easily achievable by them, or both. Moors also observes, surely correctly, that if we are prepared to accept that cognitive appraisals can be fast and automatic, then there is no reason why we should not allow the same for goal-directed thinking. So, we can accept Moors's theory of goal-directed emotional behavior while still acknowledging that in emotional episodes people act impetuously and unconsciously in choosing how to respond.

I suspect that Moors is pushing at an open door in seeking to persuade psychologists and philosophers of emotions that emotions are, at least potentially, rational. It is interesting, however, to think about the different ways that an emotional reaction can be considered “rational.” Here I depart from a direct response to Moors in order to think a little more broadly about this very interesting question that she raises. There are two distinctions to make here. The first is between two sorts of rationality—the cognitive and the strategic. A belief is cognitively rational if it is based on good evidence, whereas a behavior is strategically rational if it is likely to bring about a desired goal. Although Moors is interested primarily in the latter, if emotional episodes are based on appraisals (which are a form of belief), then the former is of relevance too. The second distinction to make here is between two ways in which something can be deemed rational—the categorial and the evaluative. Something is categorially rational if it is potentially rational, that is, if it is a candidate for rationality. Human beliefs and behaviors (including the beliefs and behaviors involved in emotional episodes) tend to be categorially rational, unlike, for instance, sensations of pain or pleasure, which cannot be evaluated as either rational or irrational. To say that something is evaluatively rational is to go further and say not only is it a candidate for rationality but also it has achieved it. So, the beliefs and behaviors involved in emotional episodes are only cognitively and strategically rational in this stronger, evaluative sense if they successfully represent the world and move the subject closer to some valued goal. Philosopher Ronald de Sousa's (1987) book on the subject remains a useful reference point for thinking about the rationality of emotion.

Now, given the huge variety of kinds of experiences and behaviors that come under the rubric of “emotion” or “emotional episode,” it seems unwise to argue too strongly in favor of a general view that all such states are either rational or irrational in an evaluative sense. Although angry violence may sometimes be rational in the sense of moving us closer to some desired goal, it very often is not, and the same would surely be the case for innumerable episodes of desire, jealousy, resentment, self-hatred, and many other emotional states. There is also a more general point that Moors's analysis does not touch upon, namely, the possibility, surely a reality in many cases, that a subject's emotional behavior can be understood as successfully moving them towards one of their goals (and so counting as “rational” in that sense) while still seeming irrational in a broader sense, as the achievement of the goal would not truly be in the subject's interest. Addictive or compulsive behaviors of all kinds might fall into this category. The drunk whose goal is yet another drink in one sense acts “rationally” when she tearfully begs the bartender to keep serving her, because she is behaving in a way that she hopes will achieve a highly valued goal. Depending on the sensibilities of the bartender, the emotional behavior might be successful. But we would hesitate to describe such emotional behavior as rational, given the objective harm the drinker is inflicting on herself through the pursuit of her goal.

This issue puts me in mind of 18th-century Anglican clergyman and philosopher Joseph Butler's response to Thomas Hobbes's view that all human action is motivated by self-interest. One of Butler's observations was that even if people are driven by what he called their own “particular passions,” this by no means guaranteed that they were acting in their own interest. “Men daily, hourly sacrifice the greatest known interest,” Butler (1726/1970) wrote,

to fancy, inquisitiveness, love or hatred, any vagrant inclination. The thing to be lamented is, not that men have so great regard to their own good or interest in the present world, for they have not enough; but that they have so little to the good of others. (pp. 14–15)

So, to put this in the terms used by Moors, we might agree that people behave, in emotional episodes, in ways that further their own goals, but it does not follow that they are acting rationally when they do so, as the attainment of their goals might not ultimately be good for them.

One final additional perspective on what it means for a behavior to be rational can be found in the writings of the Victorian moral philosopher Henry Sidgwick, who struggled over the question of what it would mean to behave perfectly rationally, from the point of view of ethics. Egoism and altruism both seemed to him to have a claim to the title of the most rational system of ethics. The dilemma arose from the ambiguity of the term “rational” when applied to actions. On the one hand it seemed obvious that it was rational for an agent to act in pursuit of his or her own goals (this is the sense in which Moors uses the term about goal-directed emotional behavior). On the other hand, however, Sidgwick thought that a truly rational being would see things from “the point of view of the universe” rather than her own interested and partial perspective. In that second sense, then, the only truly rational action is one designed to promote the greatest good of the greatest number, setting self aside (Dixon, 2008, pp. 73–80). I wonder whether Moors's goal-directed mechanism could be adapted to include a utilitarian module of some kind.

I have strayed from psychology into the history of philosophy, but that is probably appropriate, because a theoretical contribution aiming to reframe a whole area of psychological research inevitably raises philosophical questions. Moors suggests, for instance, that the cognitive component recognized by appraisal theory be stretched so that it includes not only appraisal but also a goal-directed mechanism. I wondered whether this could be translated into the kind of statement a philosopher of emotion might make about emotions, including desires as well as beliefs. This leads to the interesting question of whether all attitudes, including emotions, can be reduced without remainder to a combination of beliefs and desires (Searle, 1983; Wollheim, 1999). And this leads to the thought I end with, which is the most fundamental one raised by Moors's article, namely, whether there is anything irreducibly emotional about emotion or whether psychology can understand emotions best purely in terms of their nonemotional component parts.

I was very struck by the analogy Moors makes, on behalf of psychological construction theory, between the composition of a complex emotional experience and the composition of the air. In both cases, according to this comparison, the complex phenomenon (emotion, air) turns out to be made up of many other components, which are mixed together and can be studied in their own right. In the case of emotional episodes, the components include appraisals, bodily arousal, subjective experience, action tendencies, and so on. In the case of the air, the components are various gases. So, Moors argues, the phenomenon people describe as an “emotional episode,” like the phenomenon known as “air,” is not a “scientific set.” The rhetorical suggestion here is that the change Moors is proposing in theorizing about emotions is analogous to the progress made in chemistry when it was realized that air was not an element but had many different components. I realize that no analogy is ever supposed to be a complete one, and so I offer some concluding thoughts about the history and the limits of the analogy in a spirit of conversation rather than of contradiction.

First, it is interesting to note that the inventor of “the emotions” himself, Thomas Brown, made a parallel appeal to the history and methods of chemistry when he first introduced the category. Brown was impressed by the recent discoveries made in chemistry including, in fact, the composition of the air, by figures such as Joseph Priestley and Antoine Lavoisier. In describing his own system as a kind of “chemistry of the mind,” Brown hoped to reinforce its scientific credentials:

The science of mind, as it is a science of analysis, I have more than once compared to chemistry, and pointed out to you and illustrated its various circumstances of resemblance. In this, too, we may hope the analogy will hold,—that, as the innumerable aggregates, in the one science, have been reduced and simplified, the innumerable complex feelings in the other will admit of a corresponding reduction and simplification. (Brown, 2010, p. 134)

Joseph Priestley (1775) himself had also used chemical analogies when thinking about the way that a variety of feelings and ideas could be combined together into one “general *complex emotion*, the component parts of which will not be easily distinguishable” and which, by their mutual association will, “at length, entirely coalesce, so as never to be separately perceived” (p. xxviii). Then in the 19th century, John Stuart Mill similarly used the comparison with chemistry to endorse a nonreductionist approach to emotions as complex compounds, with properties that were not reducible to the properties of their causes. Finally, later Victorian psychologists, notably Herbert Spencer and Alexander Bain, favored a view in which emotions were aggregates of elementary feelings and bodily sensations—the more feelings and sensations, the stronger the emotional experience (Dixon, 2003, pp. 116–120, 157–158).

What, then, is the relevance of the history of such analogies in psychology to Agnes Moors's comparison of the composition of air with the composition of emotional episodes? Looking back to the use of chemical analogies by Priestley, Brown, Mill, Spencer, and Bain suggests a couple of obvious, but possibly relevant, observations. Most obvious of all, some of the components of emotions, including appraisals, goals, and conscious experiences, unlike oxygen or nitrogen, or even the firings of nerves, cannot be weighed or measured. This is why psychology will never become a kind of mental chemistry in anything but a metaphorical sense. Second, returning to these 18th- and 19th-century texts reminded me that those who advocated a broadly “chemical” approach to emotions tended to divide into two camps according to whether they treated emotions as irreducible compounds or as physical aggregates. Priestley and Mill were in the former camp, whereas Spencer and Bain, and before them Thomas Brown, were in the latter group.

So, let me end by noting that Moors, in adopting a view of emotions as aggregates (like the air) rather than as chemical compounds (with their own irreducible properties), has aligned herself with the more reductionist “aggregate” tradition. Time will tell whether such a view will prevail, but my own instinct is that a compound approach might still be preferable, that is to say, that the emotional episode produced by the combination of its components does indeed have some higher level “chemical” properties not reducible to its “physical” component parts. I am drawn in some ways to the vision of a field of academic psychology finally liberated from the confusions embodied in two centuries of “emotions” talk, and especially from the reductionist, universalizing ambitions of “affect program” and “basic emotion” theories. But as long as there are human subjects labeling their feelings as emotional episodes, experiencing them as such, and in the process changing both the felt experience and the component processes of those episodes, as psychological construction theory holds, then there will still be a task for psychologists to undertake of describing, articulating, and explaining what it is that makes emotional episodes emotional. The pursuit of that task might perhaps involve a return not only to the tradition of thinking of emotional episodes as chemical compounds but also to the phenomenological and introspective traditions that have always tried to do justice to the distinctive felt experience of emotional states (Colombetti, 2014; Eatough & Smith, 2006; Kennedy, 2012, 2015).

## References

[cit0001] Brown T. (2010). *Thomas Brown: Selected philosophical writings* (Dixon T., Ed.). Exeter, UK: Imprint Academic.

[cit0002] Butler J. (1970). *Fifteen sermons preached at the Rolls Chapel and a dissertation on the nature of virtue* (Roberts T. A., Ed.). London, UK: SPCK. (Original work published 1726)

[cit0003] Colombetti G. (2014). *The feeling body: Affective science meets the enactive mind*. Cambridge, MA: MIT Press.

[cit0004] de Sousa R. (1987). *The rationality of emotion*. Cambridge, MA: MIT Press.

[cit0005] Dixon T. (2003). *From passions to emotions: The creation of a secular psychological category*. Cambridge, UK: Cambridge University Press.

[cit0006] Dixon T. (2008). *The invention of altruism: Making moral meanings in Victorian Britain*. Oxford, UK: Oxford University Press.

[cit0007] Dixon T. (2012). “Emotion”: The history of a keyword in crisis. *Emotion Review*, , 338–344. 23459790 10.1177/1754073912445814 PMC3573683

[cit0008] Eatough V., & Smith J. (2006). I was like a wild wild person: Understanding feelings of anger using interpretative phenomenological analysis. *British Journal of Psychology*, , 483–498. 17018185 10.1348/000712606X97831

[cit0009] Frevert U. (2011). *Emotions in history: Lost and found*. Budapest, Hungary: Central European University Press.

[cit0010] Frevert U., Bailey, U., Eitler, P., Gammerl, B., Hitzer, B., Pernau, M., … Verheyen, N. (2014). *Emotional lexicons: Continuity and change in the vocabulary of feeling 1700–2000*. New York, NY: Oxford University Press.

[cit0011] Kennedy A. (2012). Contextualising the concept of the basic emotion: An historical examination of the theories of Alexander Bain and Herbert Spencer. *History and Philosophy of Psychology*, , 36–42.

[cit0012] Kennedy A. (2015). *Conceptualising emotion in psychology: A historical analysis of a current problem* (Unpublished doctoral thesis). University of Edinburgh, Edinburgh, Scotland.

[cit0013] Leys R. (2010). How did fear become a scientific object and what kind of object is it? *Representations*, , 66–104

[cit0014] Plamper J. (2015). *The history of emotions: An introduction*. Oxford, UK: Oxford University Press.

[cit0015] Priestley J. (1775). *Hartley's theory of the human mind, on the principle of the association of ideas; with essays relating to the subject of it*. London, UK: Johnson.

[cit0016] Searle J. (1983). *Intentionality: An essay in the philosophy of mind*. Cambridge, UK: Cambridge University Press.

[cit0017] Wassmann C. (2016). Forgotten origins, occluded meanings: Translation of emotion terms. *Emotion Review.*Advance online publication.

[cit0018] Watt Smith T. (2015). *The book of human emotions*. London, UK: Profile.

[cit0019] Wierzbicka A. (1999). *Emotions across languages and cultures: Diversity and universals* Cambridge, UK: Cambridge University Press.

[cit0020] Wierzbicka A. (2010). On emotions and on definitions: A response to Izard. *Emotion Review*, , 379–380.

[cit0021] Wollheim R. (1999). *On the emotions*. New Haven, CT: Yale University Press.

